# Transcriptome of sessile serrated adenoma/polyps is associated with MSI‐high colorectal cancer and decreased expression of CDX2

**DOI:** 10.1002/cam4.4810

**Published:** 2022-05-10

**Authors:** Daisuke Ohki, Nobutake Yamamichi, Yoshiki Sakaguchi, Yu Takahashi, Natsuko Kageyama‐Yahara, Mitsue Yamamichi, Chihiro Takeuchi, Yosuke Tsuji, Yasuhiro Sakai, Kouhei Sakurai, Shuta Tomida, Kazuhiko Koike, Mitsuhiro Fujishiro

**Affiliations:** ^1^ Department of Gastroenterology, Graduate School of Medicine The University of Tokyo Tokyo Japan; ^2^ Department of Joint Research Laboratory of Clinical Medicine Fujita Health University School of Medicine Aichi Japan; ^3^ Center for Comprehensive Genomic Medicine Okayama University Hospital Okayama Japan

**Keywords:** CDX2, comprehensive gene expression analysis, microsatellite instability‐high colorectal cancer, sessile serrated adenoma/polyp, transcriptome analysis

## Abstract

The objective of this study was to elucidate the molecular background of sessile serrated adenoma/polyp (SSA/P) endoscopically resected with comprehensive gene expression analysis. Gene expression profiling was performed for 10 tumor‐normal pairs of SSA/P. Cluster analysis, gene set enrichment analysis (GSEA), and consensus molecular subtype (CMS) classification of colorectal cancer (CRC) were applied to our transcriptome analysis. Unsupervised cluster analysis showed that the gene expression profile of SSA/Ps is different from that of adjacent normal epithelial cells, even in the very early stage of tumorigenesis. According to the CMS classification, our microarray data indicated that SSA/Ps were classified as CMS1. GSEA demonstrated a strong association between SSA/P and microsatellite instability‐high (MSI‐H) CRC (*p* < 10^−5^). Transcriptome analysis of five MSI‐related genes (*MSH2*, *MSH6*, *MLH1*, *PMS1*, and *PMS2*) and five CRC‐related genes (*BRAF*, *KRAS*, *APC*, *TP53*, and *CDX2*) showed that *CDX2* expression was most severely decreased in SSA/P. Immunohistochemical staining confirmed that CDX2 protein was reduced compared with the surrounding mucosa. Direct sequencing of *the BRAF* gene showed that the BRAF V600E mutation was detected in only nine of 36 cases. In a mouse model, *BRAF*, *APC*, or *CDX2* deficiency indicated that the gene expression pattern with loss of CDX2 is more similar to our SSA/Ps compared with those induced by *BRAF* or *APC* mutation. Transcriptome analysis of SSA/Ps showed characteristic gene expression with a strong resemblance to MSI‐H CRC. Downregulation of CDX2 expression is an essential molecular mechanism involved in the initial stage of SSA/P tumorigenesis. (UMIN000027365).

## Graphical abstract

SSA/Ps showed characteristic gene expression with a strong resemblance to microsatellite instability‐high colorectal cancer. And the downregulation of CDX2 expression is an essential molecular mechanism involved in the initial stage of SSA/P tumorigenesis.

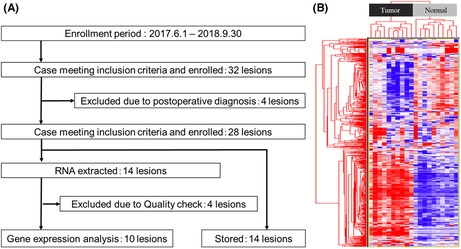

## 1 INTRODUCTION

Colorectal cancer (CRC) is a major cause of cancer‐related deaths worldwide.^1^ Based on current understanding, CRCs are derived from three different pathways: the chromosomal instability pathway, familial pathway, and serrated pathway.^2^, ^3^, ^4^ Approximately 20–30% of all CRCs are derived from serrated pathways.^4^, ^5^

Serrated lesions were classified into five groups according to the World Health Organization revised in 2019: sessile serrated lesions, sessile serrated lesions with dysplasia, hyperplastic polyps (HPs), traditional serrated adenomas (TSAs), and unclassified serrated adenomas.^6^ In Japan, serrated lesions are classified into three groups according to the Japanese Classification of Colorectal Carcinoma: sessile serrated adenoma/polyps (SSA/Ps), HPs, and TSAs.^7^

Recently, SSA/Ps have been attracting attention and are considered precursor lesions of CRC via the serrated pathway. SSA/Ps were hypothesized to have a high *BRAF* mutant and CpG island methylator phenotype. Some SSA/Ps then acquire microsatellite instability (MSI) through MLH1 methylation, leading to MSI‐high (MSI‐H) cancer, while others lead to microsatellite stable (MSS) cancer.^4^, ^8^, ^9^, ^10^, ^11^

However, the prevalence of SSA/Ps might be lower than that in tubular adenomas.^4^, ^12^, ^13^, ^14^, ^15^, ^16^ A prospective study reported that the prevalence of SSA/Ps in Japan is approximately 5%.^17^ Therefore, little is known about the molecular biological background and carcinogenesis mechanisms of SSA/Ps.

Consensus molecular subtypes (CMS) have recently gained attention.^18^, ^19^ CMS is a gene expression‐based subtype of CRCs. CRCs are classified into four subtypes: CMS1 (microsatellite instability immune, 14%), hypermutated, microsatellite unstable, and strong immune activation; CMS2 (canonical, 37%), epithelial, marked WNT and MYC signaling activation; CMS3 (metabolic, 13%), epithelial and evident metabolic dysregulation; CMS4 (mesenchymal, 23%), prominent transforming growth factor‐β activation, stromal invasion, and angiogenesis. Treatment responsiveness and prognosis differ depending on the type of CMS.

In this context, we investigated the molecular biological background of SSA/Ps through a bioinformatic analysis of comprehensive gene expression and performed CMS classification of SSA/Ps using the obtained gene expression profiles. Furthermore, based on transcriptome analysis, we investigated the molecular mechanism of the very early stages of SSA/P tumorigenesis.

## 2 METHODS

### 2.1 Patients

This study was a prospective, single‐center study. Patients with SSA/P who underwent endoscopic resection at the University of Tokyo Hospital between June 2017 and September 2018, were enrolled. All patients were endoscopically or pathologically diagnosed with SSA/P before enrollment. The following patients were excluded: a past medical history of lower gastrointestinal surgery, comorbid malignant disease, familial polyposis, inflammatory bowel disease, ethical considerations, and not being diagnosed with SSA/P pathologically. Written informed consent was obtained from all the patients. Formalin‐fixed paraffin‐embedded samples of 39 endoscopically resected SSA/Ps at our institute were also collected retrospectively for histochemical analysis.

This study was approved by the Research Ethics Committee of the Graduate School of Medicine and Faculty of the University of Tokyo and registered in the University Hospital Medical Network Clinical Trial Registry (UMIN000027365).

### 2.2 Sample preparation

Specimens were obtained from the tumor and paired with the surrounding mucosa through endoscopic biopsy immediately before resection of the target lesions using endoscopic biopsy forceps (Radial Jaw 4 PEDIATRIC 2.0 mm; Boston Scientific). The samples were immediately placed in Eppendorf tubes with RNAlater (Thermo Fisher Scientific, San Diego, CA, USA) and stored at room temperature for several hours before storing at −80°C. The SSA/Ps were endoscopically resected after biopsy and pathologically evaluated after formalin fixation. The resected tissues were re‐evaluated using endoscope photographs and pathological specimens.

### 2.3 RNA isolation and quality check (QC)

Specimens were homogenized using a Power Masher (Nippi Inc.) with BioMasher II (Nippi Inc.) in QIAzol Lysis Reagent (Qiagen). Total RNA was extracted using the miRNeasy Mini Kit (Qiagen). Quantity and purity (A260/280 ratio, A260/A230 ratio) of the extracted RNA were measured using a NanoDrop1000 spectrometer (NanoDrop Technologies). Quality check of extracted RNA was performed by measuring RNA Integrity Number and the 28S/18S rRNA ratio using an Agilent 2100 BioAnalyzer (Agilent Technologies).

### 2.4 Gene expression profiling

Oligonucleotide microarray (Agilent SurePrint G3 Human Gene Expression 8 × 60 k v3; Agilent, Inc.) was performed using 10 SSA/P‐normal pairs, followed by fluorescence assessment using a microarray scanner D (Agilent Technologies, Inc.). Raw data were logged (base2) and quantile normalization was performed using the Gene Spring software (Agilent Technologies). Raw data are registered in NCBI's Gene Expression Omnibus and are accessible through GEO Series accession number GSE198692.

### 2.5 Gene expression analysis and cluster analysis

Average linkage hierarchical clustering of both genes and cases was performed using median centering and normalization with Cluster 3.0 (http://rana.lbl.gov/EisenSoftware.htm). The results were displayed using TreeView software (http://rana.lbl.gov/EisenSoftware.htm).

### 2.6 CMS classification

Classification of gene expression profiles of SSA/Ps into the four CMSs of CRC was performed using pre‐validated multiplatform analysis packages: CMS classifier (https://github.com/Sage‐Bionetworks/CMSclassifier)^18^ and CMS caller (https://github.com/Lothelab/CMScaller).^20^

### 2.7 Direct Sanger sequencing for BRAF

Genomic DNA was extracted from tissue samples using MagMAX™ FFPE DNA/RNA Ultra Kit (Thermo Fisher Scientific, Wilmington, DE, USA) according to the manufacturer's instructions. BRAF exon 15 was amplified by PCR using the forward primer sequence 5’‐TCATAATGCTTGCTCTGATAGGA‐3′ and the reverse primer sequence 5’‐GGCCAAAAATTTAATCAGTGGA‐3′ to yield an amplicon size of 224 bp. The PCR mix contained 1x PCR Buffer for KOD FX Neo, 0.4 mM dNTPs, 0.3 μM primers, 0.5 U KOD FX Neo (TOYOBO, Osaka, Japan), and 1 μl DNA template in a total reaction volume of 25 μl. The PCR cycling conditions were as follows: 94°C for 2 min, 45 cycles of denaturation at 98°C for 10 s, annealing at 60 °C for 30 s, and extension at 68 °C for 30 s with a final extension at 68°C for 7 min. PCR products were loaded on an agarose gel, and the band corresponding to the BRAF exon‐15 fragment was excised from the gel and purified using a QIAGEN QIAquick gel extraction kit (Qiagen, Hilden, Germany). PCR products (20 ng) were used as templates for sequencing. Sequences were outsourced, and the products were analyzed by Fasmac Japan (Kanagawa, Japan).

### 2.8 Evaluation of CDX2 expression in SSA/P by immunohistochemical staining

To examine the expression of CDX2 proteins, immunohistochemical staining was performed on 39 SSAP samples, three melanoma samples, and three advanced CRC samples. Two micrometer thick sections of formalin‐fixed embedded samples were deparaffinized. Antigen activation treatment was performed at 95°C for 20 min using citrate buffer (Code.RM102‐C; LSI medience). The sections were washed with running water and immersed in TBS. Primary immunostaining with CDX2 (CMC‐235R14RUO‐0.1Ml; cosmobio.co.jp) at a 1:200 dilution was applied for 60 min at room temperature. Sections were then incubated with the secondary amino acid polymer reagent Histofine Simple Stain MAX PO (414154F; Nichirei Biosciences) for 30 min at room temperature. The reaction products were visualized by incubating the sections in a 20 mg/dL 3,3′‐diaminobenzidine tetrahydrochloride solution containing 0.006% H_2_O_2_ for 5 min. The immunostained sections were evaluated and scored by two expert pathologists.

### 2.9 GSEA of SSA/P

The gene expression profile of our SSA/Ps was compared with the gene expression profile of the mucosa of the colon of mice obtained from GSE84650 of the GEO series^21^ using Gene Set Enrichment Analysis (GSEA 4.1.0) (http://software.broadinstitute.org/gsea/). The gene expression profiles obtained from mice were as follows: a) CDX2 null mutant colon epithelium, b) BRAF V600E mutant colon epithelium, c) CDX2 null / BRAF V600E mutant colon epithelium, and d) APC mutant colon epithelium, and normal colon epithelium. GSE84650 uses GPL17400 as the platform; therefore, the HUGO Gene Nomenclature Committee (https://www.genenames.org/) was used to convert the mouse gene symbol into a human gene symbol. Using the gene expression profile converted to the human gene symbol, each mutant colon epithelium was compared with the normal colon mucosa.

Each gene set, such as (a) [Ohki mouse CDX2‐], (b) [Ohki mouse BRAF‐], (c) [Ohki mouse CDX2‐/BRAF‐], and (d) [Ohki mouse APC‐], was made by genes that were elevated compared with the normal colon. Four gene sets were included in the pre‐validated curated C2 gene set database, GSEA was performed using the gene expression profile of SSA/P obtained from the present microarray.

GSEA was then performed to analyze genes with differences in expression between tumor and paired surrounding mucosa and to characterize SSA/P by comparing the obtained gene sets to known disease‐related gene sets, especially the pre‐validated curated C2 gene set database. The [OHKI SSAP UP] gene set comprised 100 genes that were most prominently upregulated in SSA/P compared with the surrounding normal mucosa. The [OHKI SSAP DOWN] gene set was made of 100 genes that most prominently downregulated gene transcripts in SSA/P compared with the surrounding normal mucosa. Two gene sets were included in the pre‐validated curated C2 gene set database, and cross‐reference gene enrichment analysis between SSA/P, adenoma, and MSI‐H CRC was performed.

### 2.10 Biological pathway analysis

Biological pathway analysis was performed to determine functional gene ontology terms specific to tumors and paired surrounding mucosa using DAVID Bioinformatics Resources 6.7 (http://david.ncifcrf.gov/home.jsp).

## 3 RESULTS

### 3.1 Patient characteristics

**FIGURE 1:**
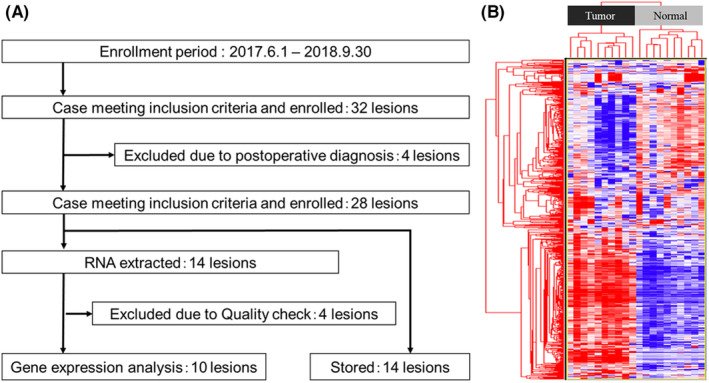
Flowchart and results of gene expression analysis between matched SSA/P and normal mucosa. (A) Flowchart of study UMIN000027365. (B) A hierarchical cluster tree of the unsupervised analysis for 694 probes satisfying the mean + 3SD from all 58,201 probes

A total of 32 cases and the surrounding mucosa were prospectively accumulated during the study period. Four cases were excluded, which were not pathologically diagnosed as SSA/P, and 28 SSA/P cases were finally included in the analysis (Figure 1A). The baseline characteristics of SSA/Ps are shown in Table S1. 67.8% (19/28) were female and 89.3% (25/28) of SSA/P lesions were located in the proximal colon. Of the 28 cases enrolled, total RNA was extracted from 14 tumors and the surrounding mucosa, in which 10 cases that passed the quality check of RNA were used for microarray gene expression analysis and bioinformatic analysis.

### 3.2 Gene expression analysis and cluster analysis

Comprehensive gene expression was evaluated, and 50 gene transcripts most prominently upregulated and downregulated in SSA/Ps (compared with the surrounding normal mucosa) are shown in Tables 1 and 2. Various genes previously reported to increase transcription have been detected, including *ANXA10*, *TM4SM4*, *VSIG1*, *SULT1C2*, *CDH3*, *KLK7*, *TFF2*, *REG4*, *SERPINB5*, *KLK11*, *MUC5AC*, *TFF1*, *MUC17*, *CTSE*, *CLDN1*, and *S100P*.^22^, ^23^, ^24^, ^25^ The top 500 gene transcripts upregulated and downregulated in SSA/Ps are also shown in Tables S2 and S3.

**TABLE 1:** Top 50 most prominently upregulated gene transcripts in SSA/P compared with the surrounding normal mucosa

Up‐regulated in SSA/Ps		Expression value (Log base2)		
Rank	Gene symbol	Ave. of tumor	Ave. of normal	Folds
1	ANXA10	11.87	1.97	9.90
2	TM4SF4	12.14	2.77	9.38
3	VSIG1	10.80	2.25	8.55
4	KLK8	9.75	1.78	7.97
5	SEMG1	8.95	1.65	7.31
6	SULT1C2	10.35	3.13	7.22
7	CDH3	9.99	2.80	7.20
8	GJB4	9.66	2.54	7.13
9	KLK7	8.59	1.49	7.11
10	TFF2	13.80	6.71	7.09
11	REG4	14.83	8.19	6.64
12	EPHX4	8.81	2.21	6.60
13	FOXD1	8.43	2.17	6.25
14	GJB5	8.29	2.04	6.25
15	MUC6	7.78	1.55	6.23
16	DPCR1	7.93	1.74	6.19
17	SERPINB5	13.23	7.25	5.98
18	SLC6A14	8.53	2.57	5.96
19	TRIM29	9.71	3.89	5.81
20	DUOXA2	11.23	5.43	5.80
21	KLK11	11.09	5.29	5.80
22	ALDOB	11.99	6.30	5.70
23	CLDN2	9.13	3.48	5.65
24	PSCA	10.99	5.35	5.64
25	MUC5AC	9.33	3.73	5.60
26	TFF1	17.58	11.98	5.59
27	MUC17	14.16	8.59	5.57
28	HTR1D	7.37	1.82	5.55
29	TCN1	7.75	2.24	5.50
30	TACSTD2	10.69	5.20	5.49
31	FOXQ1	11.27	5.82	5.45
32	SH3PXD2A‐AS1	10.13	4.70	5.42
33	CTSE	14.21	8.84	5.37
34	LOC101927318	7.12	1.79	5.33
35	CLDN18	7.01	1.73	5.27
36	lnc‐NUCB1‐1	13.08	7.81	5.27
37	KLHL30	7.21	1.97	5.24
38	VNN1	10.64	5.41	5.23
39	FAM25A	7.51	2.30	5.21
40	NR0B2	8.80	3.61	5.19
41	CRNDE	8.65	3.51	5.14
42	lnc‐SIK1‐2	8.65	3.55	5.10
43	CA9	6.74	1.64	5.10
44	lnc‐C10orf126–2	6.87	1.77	5.09
45	LINC00520	11.10	6.02	5.08
46	TRNP1	7.68	2.67	5.01
47	CLDN1	7.95	2.95	5.00
48	FEZF1‐AS1	6.62	1.69	4.93
49	S100P	17.28	12.38	4.90
50	PSAPL1	6.37	1.47	4.90

*Notes*: Tumor area: Mean expression levels in microarrays of 10 tumor lesions (log base 2).

Normal: Mean expression levels in microarrays of 10 normal lesions (log base 2).

Folds: Ave. of tumor ‐ Ave. of normal (log base 2).

**TABLE 2:** Top 50 most prominently downregulated gene transcripts in SSA/P compared with the surrounding normal mucosa

Down‐regulated in SSA/Ps		Expression value (Log base2)		
Rank	Gene symbol	Ave. of tumor	Ave. of normal	Folds
1	CWH43	5.74	10.21	−4.46
2	SSTR2	3.58	8.04	−4.46
3	CA1	10.29	14.40	−4.11
4	DPP10‐AS1	4.93	9.00	−4.06
5	CHGA	10.48	14.50	−4.02
6	TRPM6	7.79	11.73	−3.93
7	CLDN8	3.47	7.23	−3.76
8	LOC102723970	2.69	6.35	−3.66
9	HSPB3	4.74	8.39	−3.65
10	PDE6A	6.00	9.60	−3.60
11	BMP3	5.00	8.60	−3.60
12	GCG	6.32	9.91	−3.58
13	HTR4	4.50	8.08	−3.58
14	NUPR1L	4.18	7.65	−3.48
15	NEUROD1	2.92	6.34	−3.42
16	SLC26A2	12.09	15.49	−3.40
17	CLCA1	11.93	15.33	−3.40
18	CD177	9.47	12.86	−3.39
19	SATB2‐AS1	7.60	10.96	−3.36
20	UGT2A3	4.14	7.49	−3.34
21	LOC100422737	4.01	7.32	−3.31
22	XLOC_l2_009159	4.00	7.31	−3.31
23	AQP8	9.54	12.84	−3.29
24	lnc‐NR3C2–1	2.04	5.30	−3.25
25	BEST4	8.80	12.04	−3.24
26	SLC6A4	3.45	6.68	−3.23
27	WISP2	3.60	6.82	−3.22
28	SLC30A10	6.51	9.65	−3.14
29	EYA2	3.45	6.56	−3.11
30	TMIGD1	8.48	11.57	−3.10
31	ASPG	4.67	7.76	−3.09
32	lnc‐LYRM7‐2	4.62	7.70	−3.08
33	CHGB	3.35	6.41	−3.06
34	LOC101927969	3.12	6.17	−3.05
35	BEST2	8.15	11.18	−3.02
36	lnc‐SOD3‐3	5.85	8.85	−3.01
37	OGDHL	2.92	5.91	−2.99
38	ADH1C	11.69	14.66	−2.97
39	lnc‐VCAN‐1	2.75	5.71	−2.96
40	BRINP3	4.67	7.63	−2.96
41	INSL5	3.70	6.65	−2.96
42	MT1H	8.38	11.34	−2.95
43	KANK4	2.55	5.50	−2.95
44	DPP10	3.33	6.27	−2.94
45	ADH1A	8.77	11.67	−2.91
46	LRRN2	7.56	10.46	−2.90
47	SLC23A1	2.85	5.74	−2.89
48	SCN9A	2.81	5.70	−2.88
49	MS4A12	7.70	10.58	−2.88
50	DMBT1	9.37	12.23	−2.86

*Notes*: Tumor area: Mean expression levels in microarrays of 10 tumor lesions (log base 2).

Normal: Mean expression levels in microarrays of 10 normal lesions (log base 2).

Folds: Ave. of tumor ‐ Ave. of normal (log base 2).

We then identified the gene probes satisfying the criterion of mean + 3 standard deviations (SD) and performed an unsupervised cluster analysis. A total of 694 gene probes were satisfied mean + 3SD from 58,201 total probes. We created a cluster tree with these identified 694 gene probes and found that the tumor and normal mucosa were separated clearly (Figure 1B). These data indicate that the transcriptome profile of SSA/P is quite different from that of adjacent normal epithelial cells, even in the very early stage of its tumorigenesis.

### 3.3 CMS classification of CRC

**FIGURE 2:**
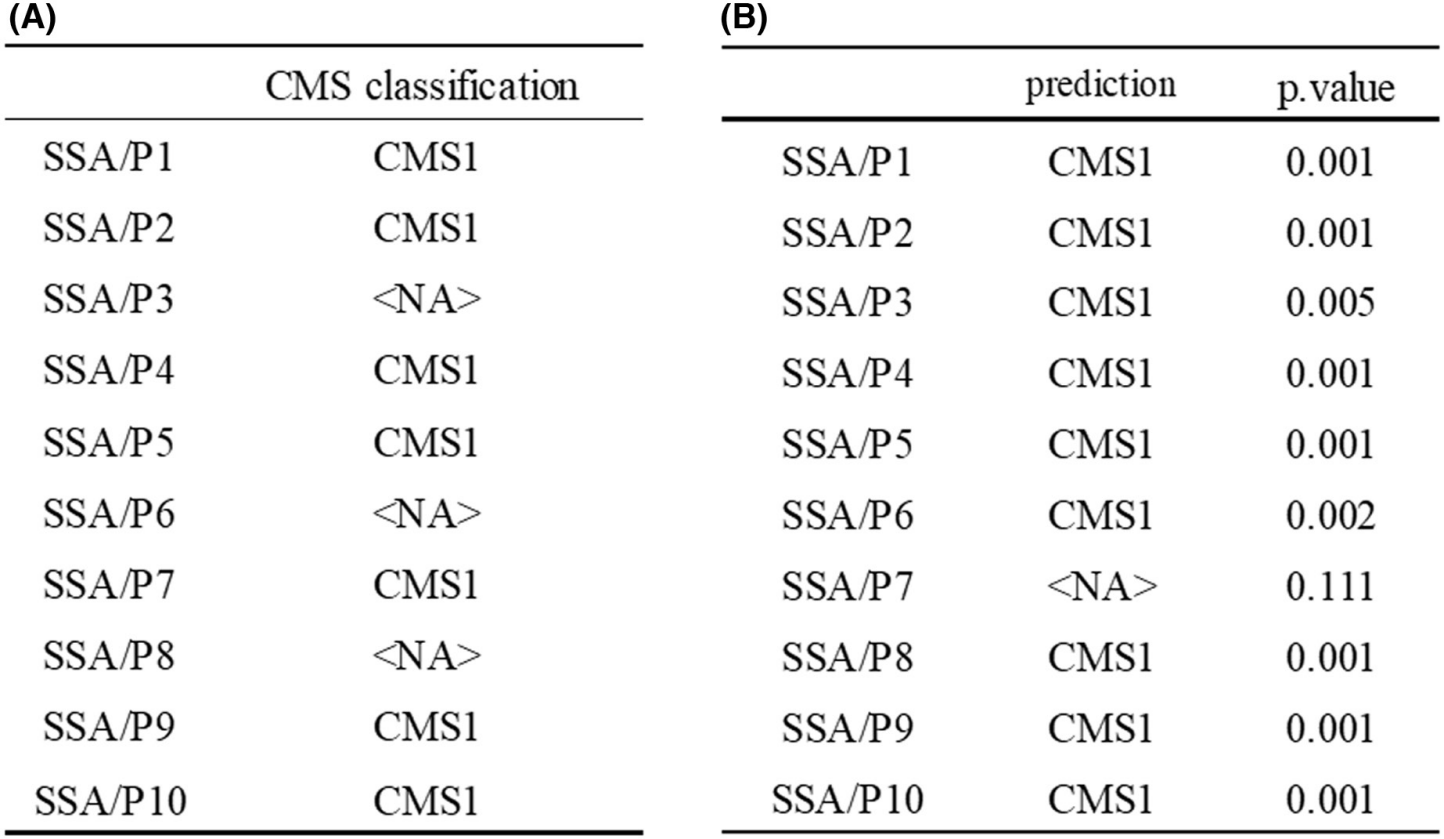
Results of CMS classification of CRC using CMS Classifier and CMS caller. CMS Classifier uses the value of “predicted CMS.” CMS Classifier and CMS caller were using pre‐validated multiplatform analysis packages. CMS Classifier (https://github.com/Sage‐Bionetworks/CMSclassifier), CMS caller (https://github.com/Lothelab/CMScaller). (A) Results of the CMS Classifier showed that 70% (7/10) of SSA/Ps were classified as CMS1. (B) Results of the CMS caller showed that 90% (9/10) of SSA/Ps were classified as CMS1. NA: not applicable

CMS classification was performed using comprehensive gene expression profiles of 10 SSA/Ps using the CMS Classifier and CMS caller. The CMS classifier showed that 70% (7/10) of SSA/Ps were classified as CMS1 (Figure 2A). The CMS caller indicated that 90% (9/10) of SSA/Ps were classified as CMS1 (Figure 2B). As a result, CMS classification analyses indicated that SSA/P is highly associated with CMS1; namely, most of the analyzed SSA/Ps were categorized as CMS1.

### 3.4 Expression of MSI‐associated genes and CRC‐related genes

Based on the results of CMS classification, we focused on the genes associated with MSI, such as *MSH2*, *MSH6*, *MLH1*, *PMS1*, and *PMS2*, using the gene expression profiles of our SSA/Ps. We also focused on *BRAF*, *KRAS*, *APC*, *TP53*, and *CDX2*, which are established cancer‐related genes important for colorectal tumorigenesis.^26^, ^27^, ^28^, ^29^, ^30^, ^31^

**FIGURE 3:**
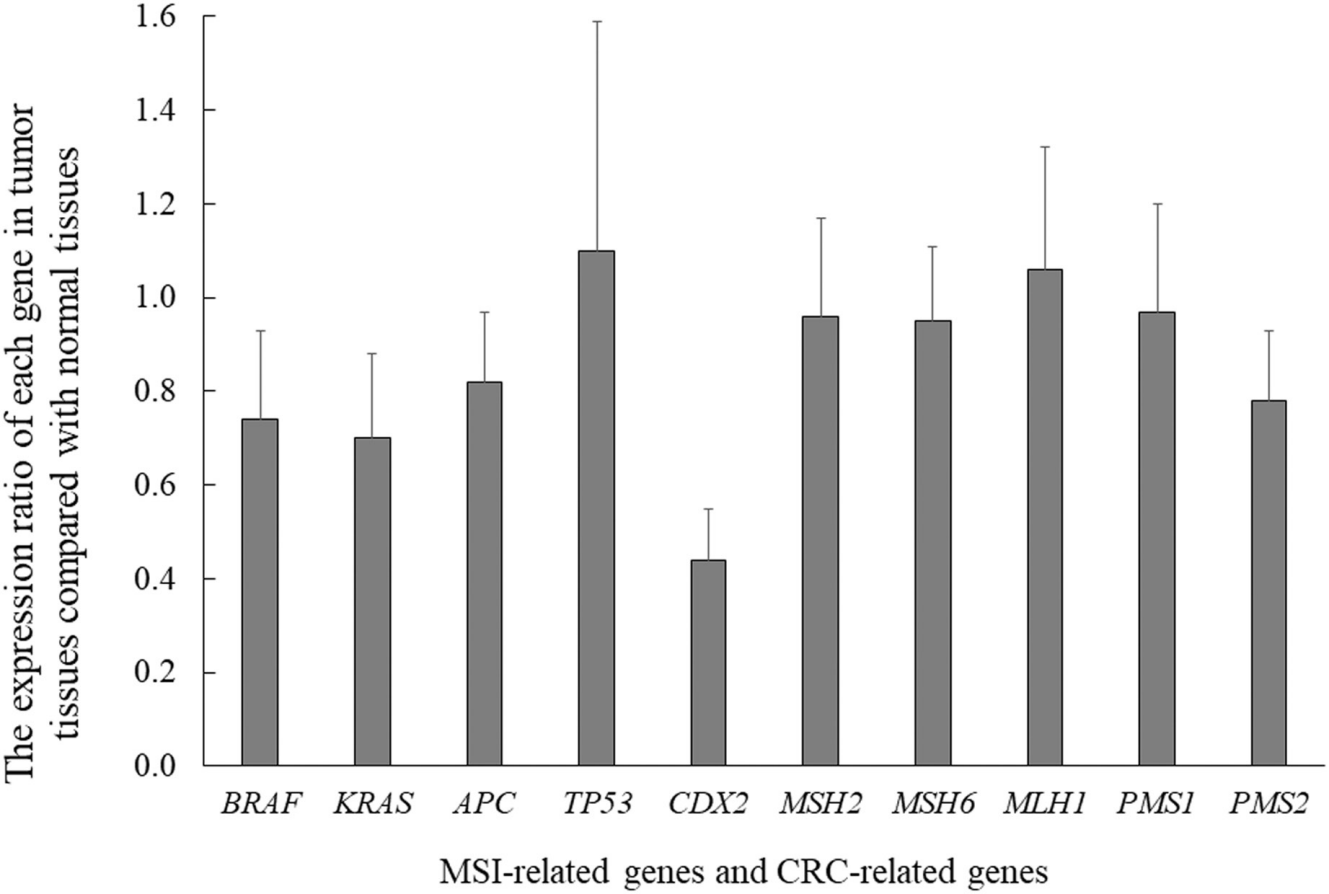
The expression ratio of MSI‐related genes and CRC‐related genes in tumor tissues compared with normal tissues. The expression ratio of each gene was as follows; MSH2 0.96, MSH6 0.95, MLH1 1.06, PMS1 0.97, PMS2 0.78, BRAF 0.74, KRAS 0.70, APC 0.82, TP53 1.10. The bar graph shows the ratio of gene expression in tumor tissues compared with that in normal tissue. Error bars represent the standard deviation of each gene

Figure 3 shows the expression ratio of each gene in tumor tissues compared with that in normal tissues. The expression ratio of MSI‐related genes in tumor tissues was not significantly different from that in normal tissues (*MSH2* 0.96, *MSH6* 0.95, *MLH1* 1.06, *PMS1* 0.97, *PMS2* 0.78). On the contrary, many cancer‐related genes tended to show decreased expression in tumor tissues (expression ratio: *BRAF* 0.74, *KRAS* 0.70, *APC* 0.82, *TP53* 1.10). Although mutation is a well‐known mechanism of oncogenesis for *BRAF*, *KRAS*, *APC*, and *TP53*, our data showed that transcription was slightly decreased for *BRAF*, *KRAS*, and *APC* (Figure 3). Among the 10 genes, the expression ratio of *CDX2* was the most severely decreased (expression ratio: 0.44). The expression level of *CDX2* was considerably lower in the tumor tissue than in the normal tissue in all 10 cases.

### 3.5 Direct Sanger sequencing for BRAF

Next, we analyzed the sequence of the *BRAF* gene since mutations in *BRAF* (especially BRAF V600E) have been most frequently reported in SSA/P.^32^, ^33^, ^34^, ^35^ Direct Sanger sequencing was performed for 38 SSA/P lesions. Two cases were excluded because of failed PCR due to the low quality of the extracted DNA. Of the 36 cases evaluated, the BRAF V600E mutation was found in nine cases. Direct Sanger sequencing indicated that 25.0% (9/36) of SSA/Ps had the BRAF V600E mutation.

### 3.6 Evaluation of CDX2 expression in SSA/P by immunohistochemistry

**FIGURE 4:**
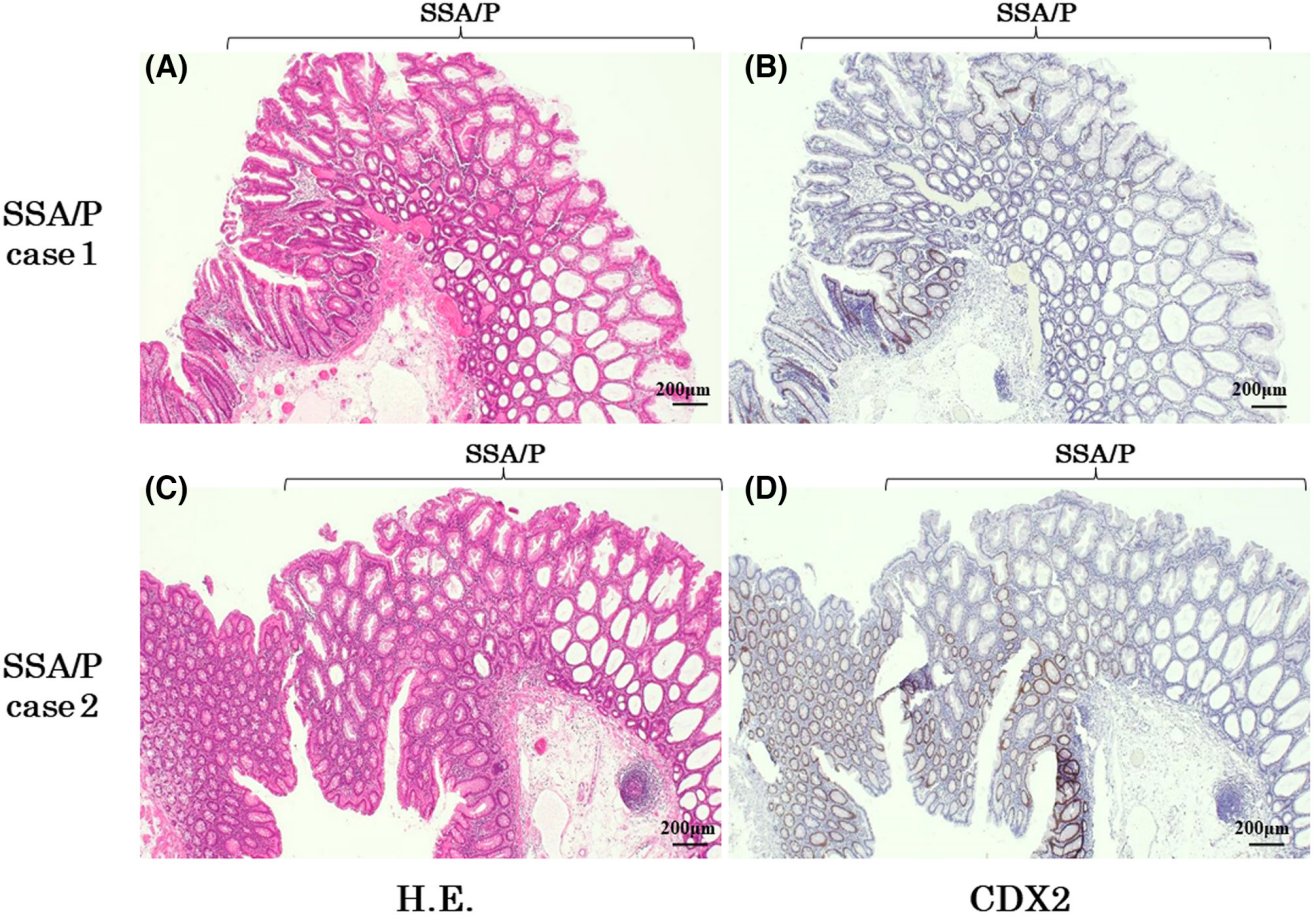
Immunohistochemical staining of CDX2 in two independent SSA/P cases. At x40 magnification, H.E. (hematoxylin–eosin) staining (A, C) demonstrates that SSA/P lesions with distorted crypts spread mostly on the surface of colorectal mucosa. Histochemical staining with anti‐CDX2 antibody (B, D) demonstrates decreased expression of CDX2 in the SSA/P tumor compared with surrounding normal colorectal epithelial cells

Of the various CRC‐related genes, our results showed that *CDX2* expression was severely decreased in tumor tissues compared with that in normal tissues (Figure 3). To confirm whether the protein level of CDX2 was decreased in SSA/Ps, immunohistochemical staining was performed on 39 cases of SSA/P. As shown in Figure 4, immunohistochemical staining clearly showed a remarkable reduction in CDX2 protein expression in the very early stages of SSA/Ps, all of which were endoscopically resected. The immunohistochemistry staining of CDX2 in 39 SSA/P samples was summarized in Table S5, in which the staining rate in SSA/P tumors was shown in comparison with the staining rate of surrounding normal colorectal mucosa.

### 3.7 Comparison of comprehensive gene expression between our SSA/Ps and a mouse model deficient in BRAF, APC, or CDX2 genes

**FIGURE 5:**
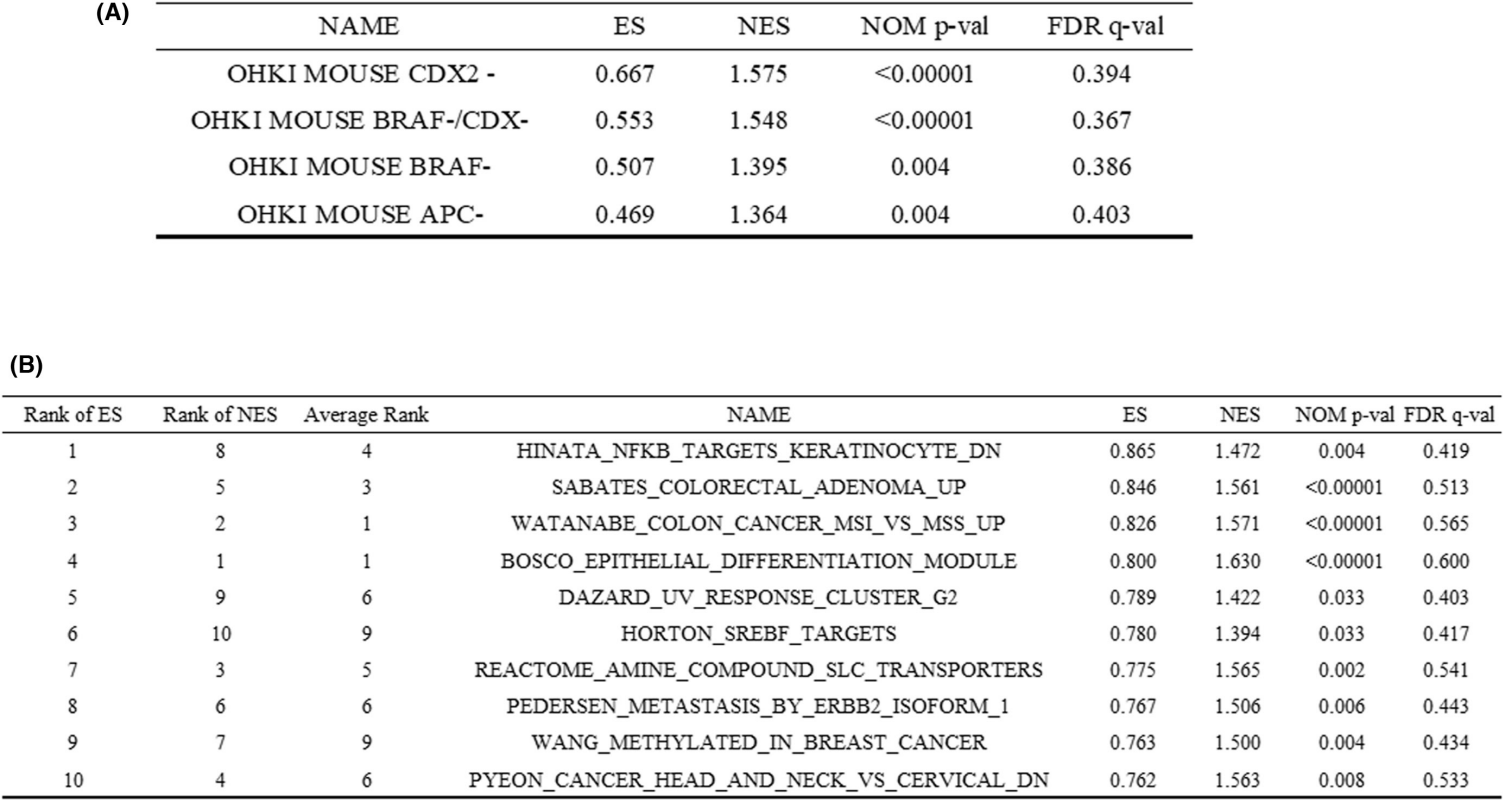
Result of GSEA. (A) Result of GSEA between our SSA/Ps and four gene sets of a model mouse deficient in *BRAF*, *APC*, or *CDX2* genes (GSE84650). The gene expression pattern induced by deletion of CDX2 is more similar to that of human SSA/Ps, compared with the gene expression pattern induced by BRAF or APC mutation. (B) Results of GSEA with C2 curated. SSA/P has a gene expression profile with a strong correlation between MSI‐H CRC and tubular adenoma (*p* < 10^−5^). ES: Enrichment score, the value indicating the degree of association with the gene set registered in GSEA. Nominal *p* < 10^−5^. OHKI MOUSE CDX2‐: CDX2 null mutant colon epithelium. OHKI MOUSE BRAF+/CDX‐: CDX2 Null / BRAF V600E mutant colon epithelium. OHKI MOUSE BRAF+: BRAF V600E mutant colon epithelium. OHKI MOUSE APC‐: APC mutant colon epithelium. Folds: tumor‐normal (log base 2)

Our analyses revealed that CDX2 expression was severely decreased in early‐stage SSA/Ps (Figures 3, 4). In addition, our results also indicated that the expression and function of BRAF and APC may be diminished (Figure 3), although the mutation of the *BRAF* gene was not as frequent (25.0%). To confirm these results in silico, we compared them with mRNA transcription data of animal models deficient in *BRAF*, *APC*, or *CDX2* (GSE84650).^21^ GSEA C2 curated with the four gene sets from GSE84650 showed that the correlation with the gene expression profile of our SSA/P was higher in the order of [Ohki mouse CDX2‐] (Enrichment score 0.667, *p* < 10^−5^), [Ohki mouse CDX2‐/BRAF‐] (Enrichment score 0.553, *p* < 10^−5^), [Ohki mouse BRAF‐] (Enrichment score 0.507, *p* = 0.008), and [Ohki mouse APC‐] (Enrichment score 0.469, *p* = 0.004) (Figure 5A). These results indicate that the gene expression pattern induced by the deletion of *CDX2* is more similar to that of human SSA/Ps, compared with the gene expression pattern induced by *BRAF* or *APC* mutations. Seven genes (*VSIG1*, *TFF2*, *FOXQ1*, *VNN1*, *PRSS22*, *IL1RN*, and *MMP7*) included in the [Ohki mouse CDX2‐] gene set were found in the top 100 genes (Table S2) that were elevated by our SSA/Ps compared with the surrounding mucosa. These results indicate that decreased expression of *CDX2* considerably influences the molecular biology of the early stage of SSA/P tumorigenesis.

### 3.8 GSEA for SSA/P

GSEA to classify the gene expression of SSA/Ps using the pre‐verified C2 curated gene set database showed that 265 gene sets were significantly upregulated in SSA/Ps (*p* < 0.05), whereas 12 gene sets were downregulated in SSA/Ps (*p* < 0.05). The top 10 gene sets upregulated in SSA/Ps are shown in Figure 5B, together with ES (enrichment score) and normalized enrichment score (NES). Various gene sets concerning tumorigenesis, epithelial differentiation/regeneration, oncogene‐related, etc. were identified. Among them, the top three gene sets deserved our attention as follows. [HINATA_NFKB_TARGETS_KERATINOCYTE_DN] (genes downregulated in primary keratinocytes by expression of p50 and p65 components of NFKB) was the most significantly correlated gene set for up‐regulation (Enrichment score 0.865, *p* = 0.004). In this gene set, 39.1% (9/23) of genes were four times higher in SSA/Ps than in the surrounding normal mucosa. [SABATES_COLORECTAL_ADENOMA] was the gene set from the colorectal adenoma reported by Sabates et al., which consists of genes upregulated in colorectal adenoma compared with surrounding normal mucosa (Enrichment score 0.846, *p* < 10^−5^).^36^ [WATANABE_COLON_CANCER_MSI_VS_MSS_UP] was the gene set derived from MSI‐high CRC reported by Watanabe et al., which comprises genes upregulated in MSI‐high CRC compared with MSS CRC (Enrichment score 0.826, *p* < 10^−5^).^37^ The specimens were obtained from advanced CRCs resected surgically, except for familial adenomatous polyposis and hereditary nonpolyposis colorectal cancer.

We applied our two gene sets, [OHKI SSAP UP] and [OHKI SSAP DOWN], into GSEA C2 curated, and cross‐reference analysis was performed with adenoma and MSI‐high CRC (Table S4). Our analyses indicated that gene expression of SSA/P was significantly associated with MSI‐H CRCs but not with colorectal adenomas.

### 3.9 Biological pathway analysis

The DAVID pathway analysis was performed using 360 probes. These probes were selected to demonstrate a twofold difference in expression between all matched tumor‐normal pairs (320 upregulated and 40 downregulated). The functional classifications upregulated in tumors are shown in Table S6. “Annexin V" was the most upregulated in the tumors. Many genes such as “Secreted” and “Signal,” which were correlated with secretion inside and outside the cell, were included. In contrast, no functional classification was detected that was significantly downregulated in tumors (Table S7).

## 4 DISCUSSION

In this study, we performed comprehensive gene expression analysis by microarray using the very early stages of SSA/P tumors. Some reports have described the transcriptome analysis of SSA/Ps in Western countries.^22^, ^23^, ^24^, ^25^ However, to the best of our knowledge, this is the first study to investigate the comprehension of bioinformatic analysis in East Asia. Based on the very high level of endoscopic therapy in Japan, all the analyzed SSA/P tumors were in the initial stages of tumorigenesis.

CMS classification by CMS Classifier^18^ and CMS caller^20^ demonstrated that SSA/Ps are classified as CMS1. These results suggest that SSA/Ps can progress to MSI‐H CRCs. With reference to gene sets actually used in CMS caller,^20^ the notable upregulated gene sets of CMS1 are “MSI‐high” and “cell cycle.” On the contrary, inactivated gene sets of CMS1 include “CDX2,” “intestinal differentiation,” and “LGR5”. “MSI‐high” gene set should reflect the identified “WATANABE_COLON_CANCER_MSI_VS_MSS_UP" showing the similarity with SSA/P (Figure 5B). Previous reports have also suggested that SSA/Ps might progress to MSI‐H CRCs based on the analysis of limited specific genes.^4^, ^25^ “Cell cycle” gene set suggests that cell division is activated in SSA/P tumorigenesis. In contrast, “CDX2,” “intestinal differentiation,” and “LGR5” should reflect the weakened characteristics of the small/large intestines in SSA/P. In our present study, we clearly showed that a decrease of CDX2 occurs in the very early stage of SSA/P tumorigenesis (Figure 4). Considering all these, we think it is reasonable that SSA/P is categorized into CMS1.

This is the first study from East Asia, where the prevalence and character of gastrointestinal disease were quite different from Western countries. It has been reported that when MLH1 deletion is added to SSA/P, it acquires heteromorphism and progresses to MSH‐H CRC.^38^ Therefore, We evaluated the expression levels of genes associated with MSI (*MSH2*, *MSH6*, *MLH1*, *PMS1*, and *PMS2*) or CRC (*BRAF*, *KRAS*, *APC*, *TP53*, and *CDX2*).^26^, ^27^, ^28^, ^29^, ^30^, ^31^ Our transcriptome analysis showed that some of them, including *BRAF* and *APC*, were mildly decreased, but direct Sanger sequencing of the *BRAF* gene showed a mutation rate (BRAF V600E) of 25%, which was less than that previously reported.^4^, ^32^ We also found that *CDX2* showed the lowest expression ratio in tumor tissues compared with normal tissues, which was verified by immunohistochemical staining.

Furthermore, GSEA using animal models (GSE84650) showed that gene expression patterns induced by loss of *CDX2* were more similar to those of our SSA/Ps, compared with those induced by *BRAF* or *APC* mutations. Some reports also showed decreased expression of *CDX2* in SSA/P,^26^, ^39^ and it has also been reported that decreased expression of *CDX2* correlates with BRAF V600E mutation and the serrated pathway.^27^ *CDX2* is a well‐known master gene essential for intestinal development, differentiation, and maintenance of function.^40^ Therefore, we speculate that decreased expression of *CDX2* plays an important role in the very early stages of SSA/P tumorigenesis.

GSEA also showed that SSA/P has a gene expression profile with a strong correlation to MSI‐H CRC and tubular adenoma. Previously, it was thought that SSA/Ps might have different characteristics from tubular adenomas, but our results suggest that SSA/Ps and tubular adenomas might have similar gene expression profiles. On the contrary, cross‐reference analysis showed that SSA/Ps were correlated with MSI‐H CRCs, but tubular adenomas were not correlated with MSI‐H CRCs.

We acknowledge that there are several limitations to our study. First, the subjects of comprehensive gene expression in this study were only SSA/Ps. In the serrated pathway, microvesicular HPs (MVHPs) are thought to be precursor lesions of SSA/Ps, and SSA/Ps acquire MSI accumulation of DNA methylation leading to MSI‐H CRCs via SSA/P with cytological dysplasia. If comprehensive gene expression analysis of each MVHP, SSA/Ps with cytological dysplasia, and MSI‐H cancers can be performed, it might be possible to obtain results approaching the mechanism of oncogenesis by comparing with the results of SSA/Ps in this study. However, the prevalence of SSA/Ps with cytological dysplasia is very low, and detection of SSA/Ps with cytological dysplasia is difficult. Furthermore, the dysplastic area of SSA/Ps with cytological dysplasia did not occupy the entire lesion, and it is difficult to correctly obtain endoscopic biopsy specimens from dysplastic areas based on endoscopic findings. We should compare SSA/Ps, SSA/Ps with cytological dysplasia, and MSI‐H CRCs and confirm whether SSA/Ps progress to MSI‐H CRCs. Second, the cases of SSA/Ps in which we performed comprehensive gene analysis were scarce. Third, we use the name “SSA/P" in this study. In 2019, the revision of the WHO classification changed the name of SSA/P to SSL. Strictly speaking, these two are not identical, but we think our results from SSA/P can mostly apply to understanding SSL based on the similarity of SSA/P and SSL. In fact, the name “SSA/P” is still widely used in the world. Fourth, we believe that it is necessary to investigate the Beta‐catenin localization and ERK‐phosphorylation in order to evaluate the functional effects of the changes in APC and BRAF transcript levels. We would like to make this an issue for future study.

In conclusion, our study demonstrates that the gene expression profiles of SSA/Ps are strongly correlated with those of MSI‐H CRCs and that downregulation of CDX2 expression is essential for the first step in the tumorigenesis of SSA/P.

## AUTHOR CONTRIBUTIONS

Conception and design: Daisuke Ohki, Nobutake Yamamichi, Shuta Tomida. Development of methodology: Daisuke Ohki, Nobutake Yamamichi, Yoshiki Sakaguchi, Yu Takahashi, Natsuko Kageyama‐Yahara, Mitsue Yamamichi, Chihiro Takeuchi. Data analysis and interpretation: Daisuke Ohki, Nobutake Yamamichi, Yoshiki Sakaguchi, Shuta Tomida, Natsuko Kageyama‐Yahara, Mitsue Yamamichi, Yasuhiro Sakai, Kouhei Sakurai. Writing, review, and/or revision of the manuscript: Daisuke Ohki, Nobutake Yamamichi, Yoshiki Sakaguchi, Shuta Tomida, Chihiro Takeuchi, Natsuko Kageyama‐Yahara, Mitsue Yamamichi, Yasuhiro Sakai, Kouhei Sakurai, Yosuke Tsuji, Kazuhiko Koike and Mitsuhiro Fujishiro.

## CONFLICT OF INTEREST

All authors have no conflict of interest.

## ETHICAL APPROVAL STATEMENT

This study was approved by the Research Ethics Committee of the Graduate School of Medicine and Faculty of the University of Tokyo (review number: 11537‐(1)).

## CLINICAL TRIAL REGISTRATION NUMBER

This study was registered in the University Hospital Medical Network Clinical Trial Registry (UMIN000027365).

## INFORMED CONSENT

Written informed consent was obtained from all the patients.

## Supporting information


Tables S1‐S7

## Data Availability

Raw data are registered in NCBI's Gene Expression Omnibus and are accessible through GEO Series accession number GSE198692.
